# Supplementary material for: Metabolic Effects of Resistant Starch Type 2: A Systematic Literature Review and Meta-Analysis of Randomized Controlled Trials
Source: Nutrients. 2019 Aug 8;11(8):1833. doi: 10.3390/nu11081833 (PMC6723691; doi:10.3390/nu11081833)
Supplement: Supplementary file 1 [file nutrients-11-01833-s001.pdf]

Supplementary Supporting Material

Supplemental Table 1. Search Strategy

Ovid Medline: 1 January 1982 to 31 December 2018
1. exp Adult/<br>2. adult<br>3. exp Humans/<br>4. human*.mp<br>5. exp Patients/<br>6. patient*.mp<br>7. 1 or 2 or 3 or 4 or 5 or 6<br>8. exp Dietary Fiber/<br>9. Dietary fib*.mp<br>10. exp Starch/<br>11. starch.mp<br>12. resistant starch.mp.<br>13. retrograded starch.mp.<br>14. high amylose starch.mp.<br>15. RS2.mp.<br>16. high amylose adj starch.mp.<br>17. HAMSRS2.mp.<br>18. HAM-RS2.mp.<br>19. 8 or 9 or 10 or 11 or 12 or 13 or 14 or 15 or 16 or 17 or 18<br>20. exp Metabolic Diseases/<br>21. metabolic dis*.mp<br>22. metabol*.mp.<br>23. gluco*.mp.<br>24. lipid.mp.<br>25. insulin resistan*.mp.<br>26. appetite.mp.<br>27. weight.mp.<br>28. body weight.mp<br>29. satiety.mp.<br>30. waist circumference.mp.<br>31. 20 or 21 or 22 or 23 or 24 or 25 or 26 or 27 or 28 or 29 or 30<br>32. 7 and 19 and 31

Supplementary Supporting Material

Supplemental Figure 1: Funnel plot of studies reporting the effects of RS2 supplementation on fasting plasma glucose (mmol/L) in healthy subjects, subjects with MetS and subjects with T2DM.

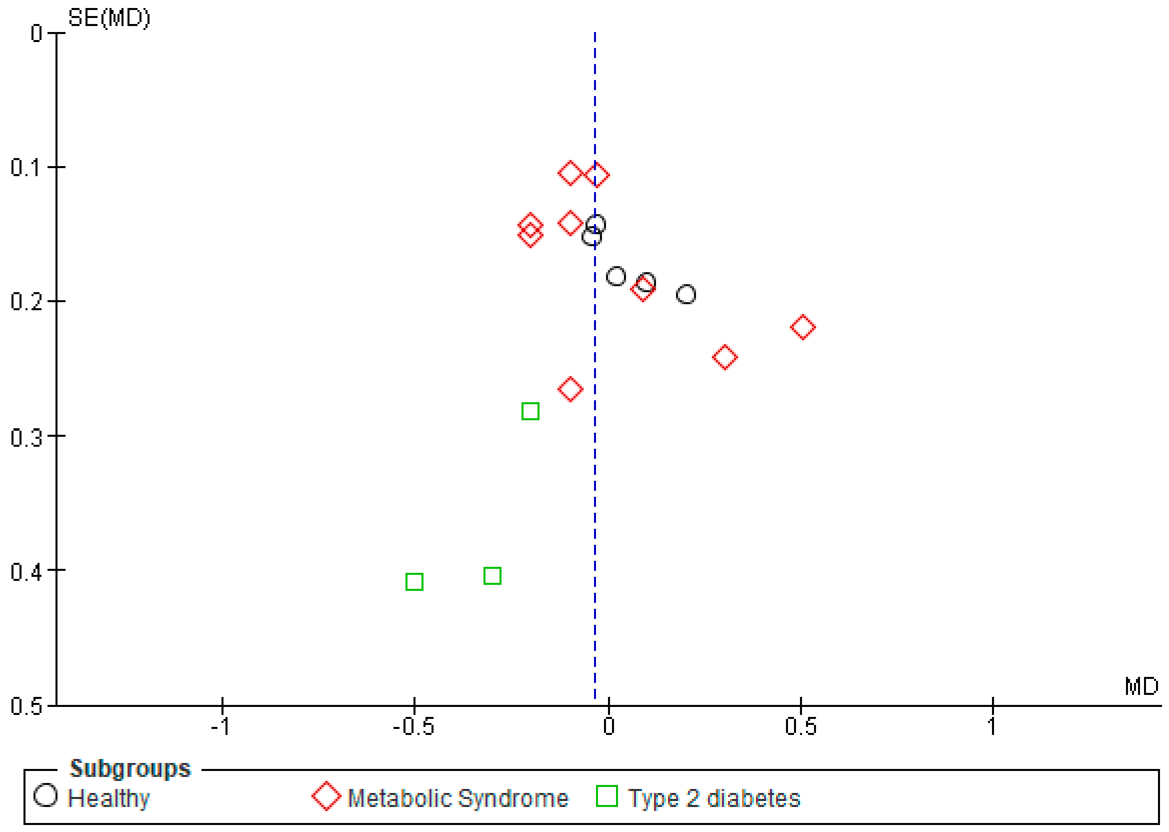

Supplementary Supporting Material

Supplemental Figure 2: Funnel plot of studies reporting the effects of RS2 supplementation on HOMA-IR in healthy subjects, subjects with MetS and subjects with T2DM.

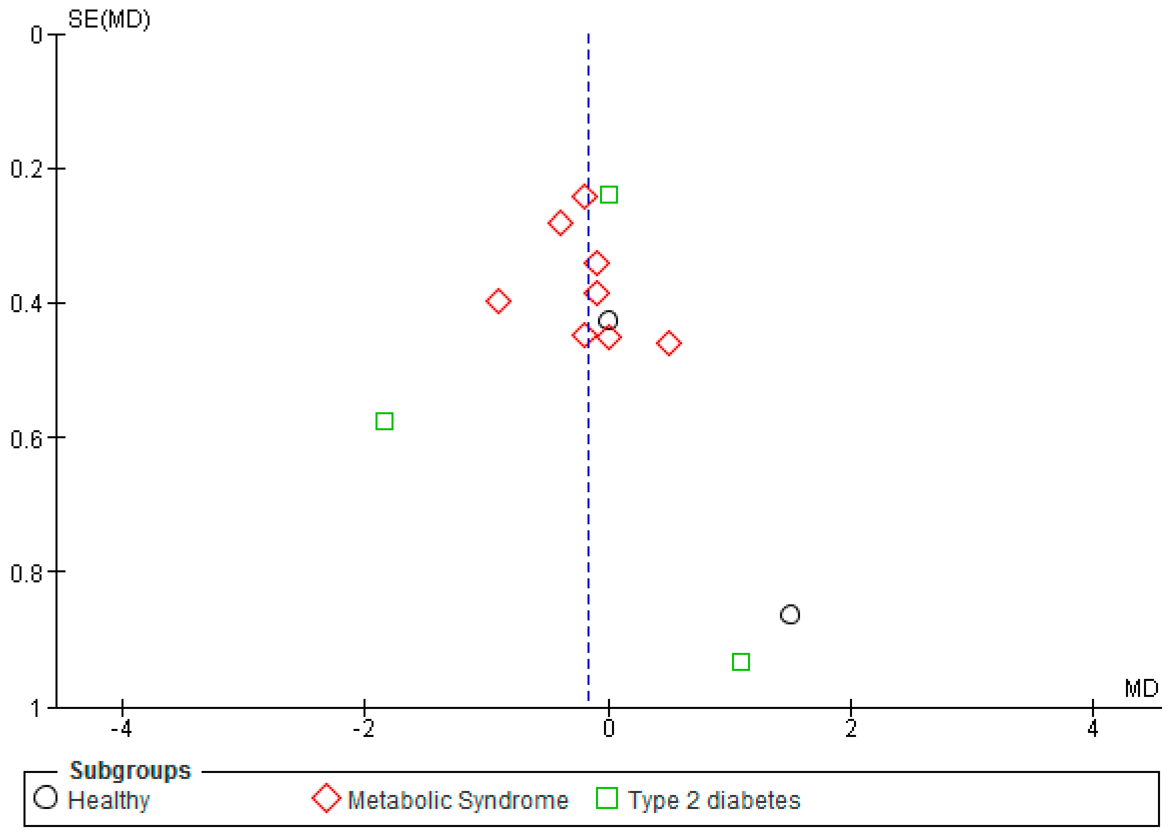

Supplementary Supporting Material

Supplemental Figure 3: Meta-regression analysis exploring the effect of moderator covariates on HOMA-IR effect-size variation between studies.

Main results for Model 1, Random effects (MM), Z-Distribution, Std diff in means

Covariate	Coefficient	Standard Error	95% Lower	95% Upper	Z-value	2-sided P-value
**Intercept**	-1.1236	2.2596	-5.5523	3.3052	-0.50	0.6190
**Age**	-0.0094	0.0256	-0.0595	0.0407	-0.37	0.7128
**Dose**	0.0075	0.0129	-0.0178	0.0328	0.58	0.5603
**Time**	-0.0541	0.0513	-0.1547	0.0465	-1.05	0.2917
**BMI**	0.0493	0.0464	-0.0416	0.1401	1.06	0.2880

Statistics for Model 1

Test of the model: Simultaneous test that all coefficients (excluding intercept) are zero

$Q = 2.86$, $df = 4$, $p = 0.5811$

Goodness of fit: Test that unexplained variance is zero

$Tau^2 = 0.0604$, $Tau = 0.2458$, $I^2 = 32.64\%$, $Q = 10.39$, $df = 7$, $p = 0.1674$

Comparison of Model 1 with the null model

Total between-study variance (intercept only)

$Tau^2 = 0.0530$, $Tau = 0.2303$, $I^2 = 30.47\%$, $Q = 15.82$, $df = 11$, $p = 0.1479$

Proportion of total between-study variance explained by Model 1

R^2 analog = 0.00 (computed value is -0.14)

Supplementary Supporting Material

Supplemental Figure 4: Funnel plot of studies reporting the effects of RS2 supplementation on total cholesterol (mmol/L) in healthy subjects, subjects with MetS and subjects with T2DM.

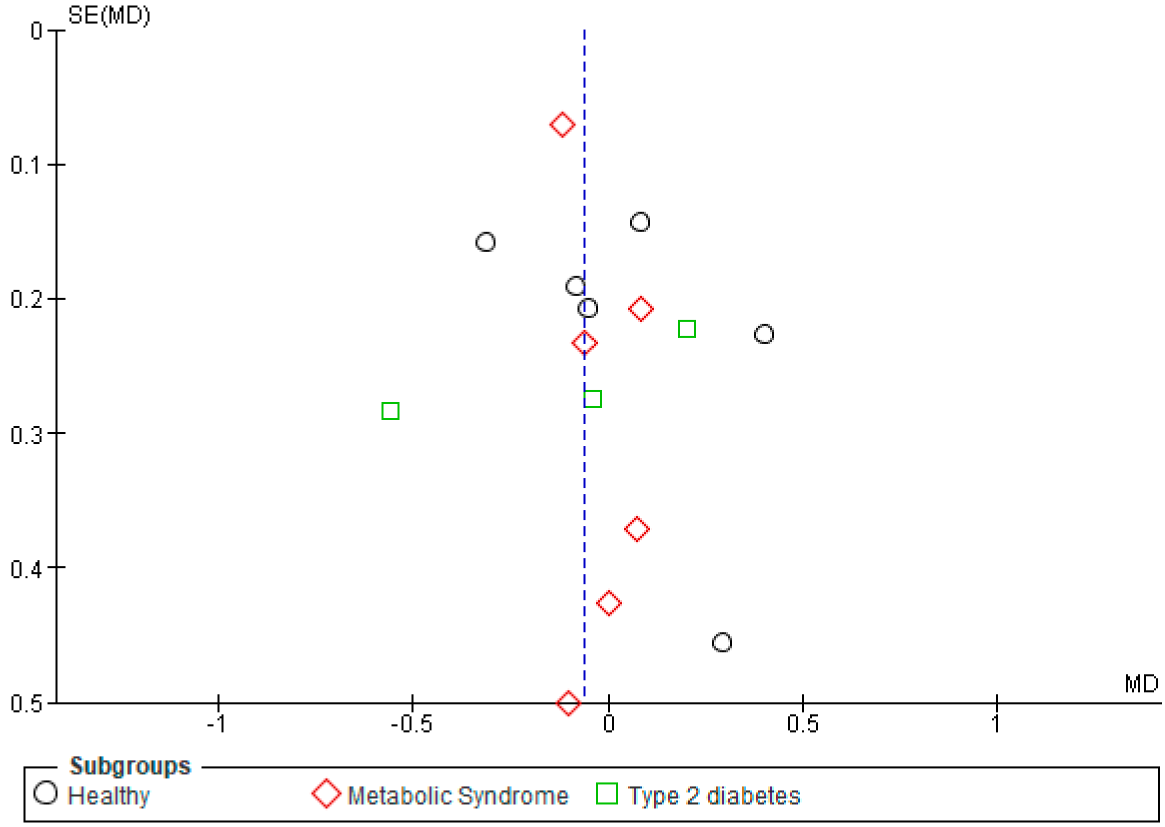

Supplementary Supporting Material

Supplemental Figure 5: Funnel plot of studies reporting the effects of RS2 supplementation on HDL-cholesterol (mmol/L) in healthy subjects, subjects with MetS and subjects with T2DM.

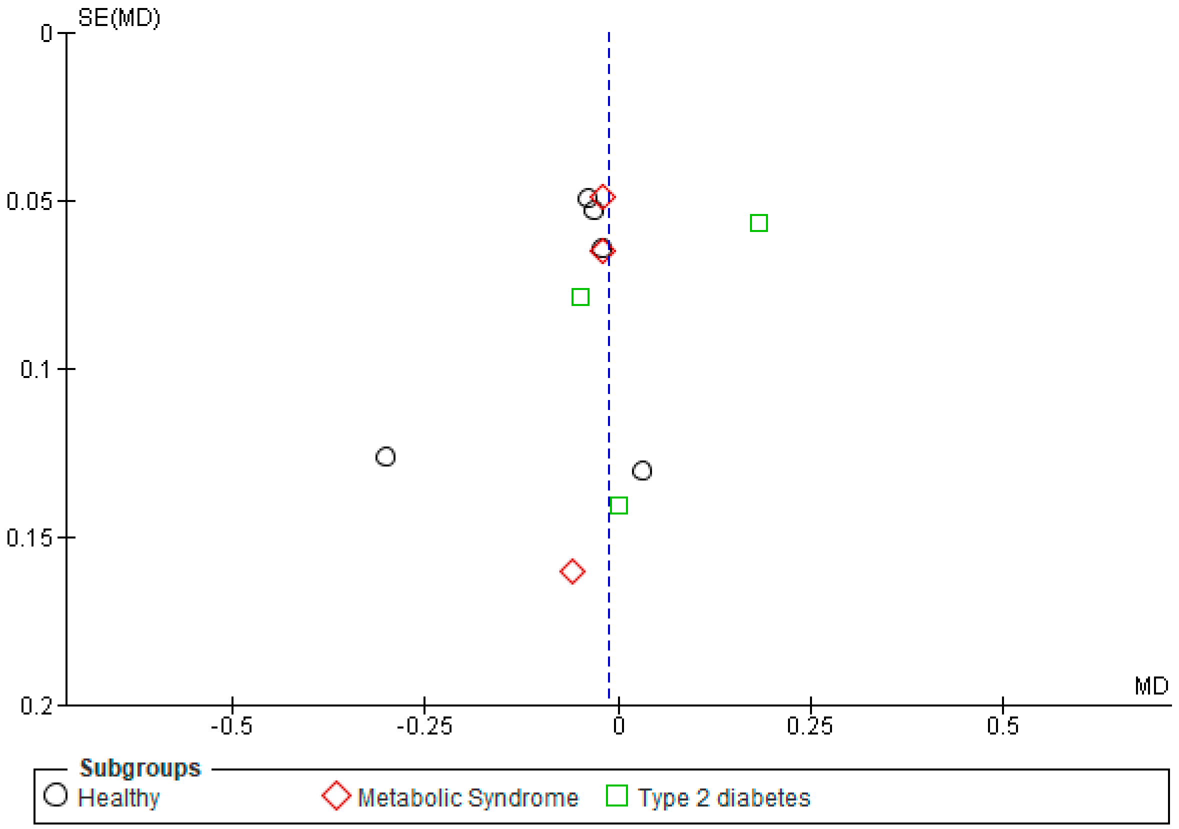

Supplementary Supporting Material

Supplemental Figure 6: Funnel plot of studies reporting the effects of RS2 supplementation on TAG (mmol/L) in healthy subjects, subjects with MetS and subjects with T2DM.

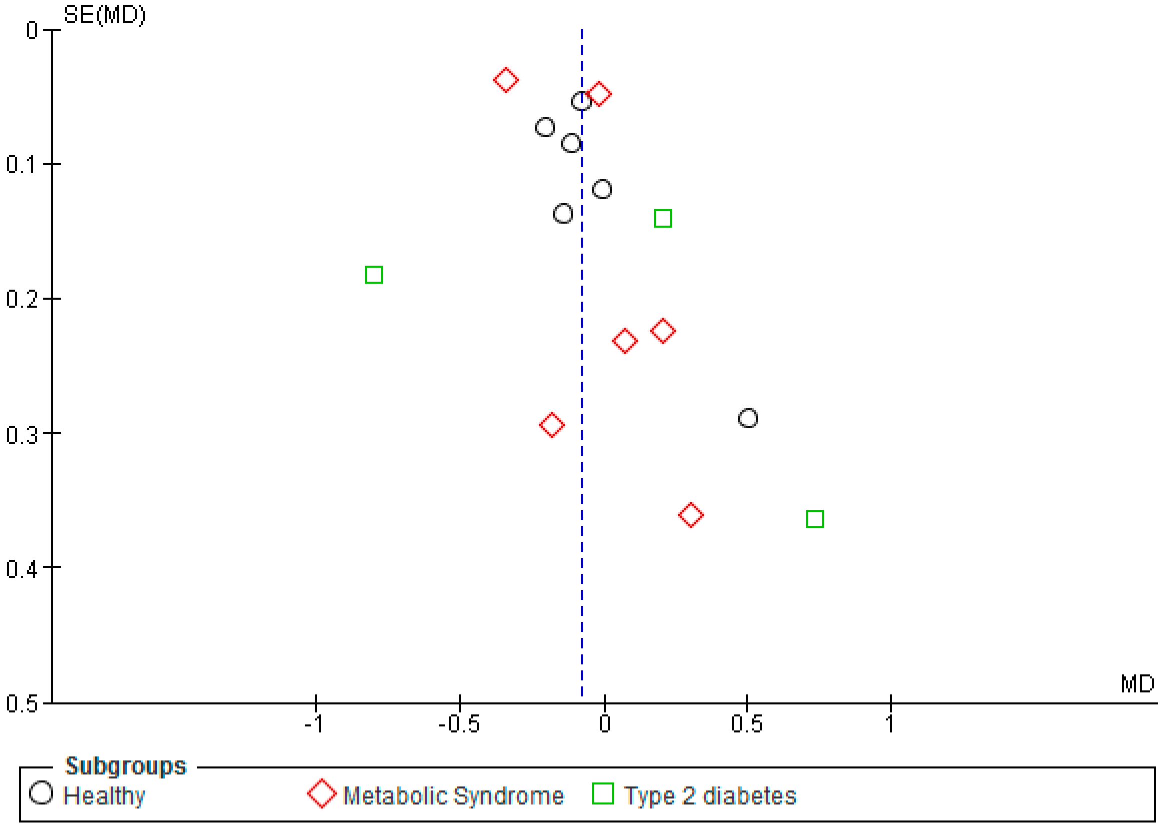

Supplementary Supporting Material

Supplemental Figure 7: Meta-regression analysis exploring the effect of moderator covariates on TAG effect-size variation between studies.

Main results for Model 1, Random effects (MM), Z-Distribution, Std diff in means

Covariate	Coefficient	Standard Error	95% Lower	95% Upper	Z-value	2-sided P-value
**Intercept**	-2.5619	1.0797	-4.6781	-0.4457	-2.37	0.0177
**Age**	-0.0454	0.0203	-0.0853	-0.0056	-2.24	0.0253
**BMI**	0.1632	0.0583	0.0490	0.2774	2.80	0.0051
**Time**	-0.0912	0.0506	-0.1903	0.0080	-1.80	0.0717

Statistics for Model 1

Test of the model: Simultaneous test that all coefficients (excluding intercept) are zero

Q = 8.82, df = 3, p = 0.0317

Goodness of fit: Test that unexplained variance is zero

$Tau^2 = 0.0630$, Tau = 0.2509, $I^2 = 37.64\%$, Q = 11.23, df = 7, p = 0.1291

Comparison of Model 1 with the null model

Total between-study variance (intercept only)

$Tau^2 = 0.1480$, Tau = 0.3846, $I^2 = 62.29\%$, Q = 26.52, df = 10, p = 0.0031

Proportion of total between-study variance explained by Model 1

R^2 analog = 0.57

R^2 for Model 1, Random effects (MM), Z-Distribution, Std diff in means

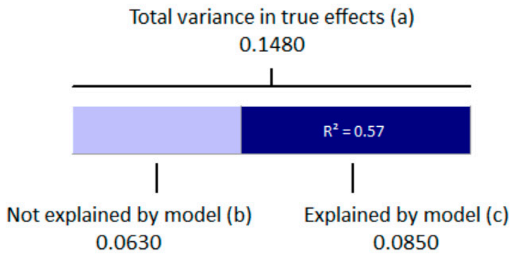

$$R^2 = \frac{\text{Explained (c)}}{\text{Total (a)}} = \frac{0.0850}{0.1480} = 0.57$$

(a) To compute the total variance (of all studies about the grand mean) we run the regression with no covariates.
(b) To compute the variance not explained by the model (of all studies about the regression line) we run the regression with the covariates.
(c) The difference between these values gives us the variance explained by the model.
